# N439K Variant in Spike Protein Alter the Infection Efficiency and Antigenicity of SARS-CoV-2 Based on Molecular Dynamics Simulation

**DOI:** 10.3389/fcell.2021.697035

**Published:** 2021-08-03

**Authors:** Wenyang Zhou, Chang Xu, Pingping Wang, Meng Luo, Zhaochun Xu, Rui Cheng, Xiyun Jin, Yu Guo, Guangfu Xue, Liran Juan, Anastasia A. Anashkina, Huan Nie, Qinghua Jiang

**Affiliations:** ^1^School of Life Science and Technology, Harbin Institute of Technology, Harbin, China; ^2^Key Laboratory of Biological Big Data (Harbin Institute of Technology), Ministry of Education, Harbin, China; ^3^Engelhardt Institute of Molecular Biology, Russian Academy of Sciences, Moscow, Russia

**Keywords:** SARS-CoV-2, N439K, molecular dynamics, binding free energy, neutralizing antibody

## Abstract

Severe acute respiratory syndrome coronavirus 2 (SARS-CoV-2), causing an outbreak of coronavirus disease 2019 (COVID-19), has been undergoing various mutations. The analysis of the structural and energetic effects of mutations on protein-protein interactions between the receptor binding domain (RBD) of SARS-CoV-2 and angiotensin converting enzyme 2 (ACE2) or neutralizing monoclonal antibodies will be beneficial for epidemic surveillance, diagnosis, and optimization of neutralizing agents. According to the molecular dynamics simulation, a key mutation N439K in the SARS-CoV-2 RBD region created a new salt bridge with Glu329 of hACE2, which resulted in greater electrostatic complementarity, and created a weak salt bridge with Asp442 of RBD. Furthermore, the N439K-mutated RBD bound hACE2 with a higher affinity than wild-type, which may lead to more infectious. In addition, the N439K-mutated RBD was markedly resistant to the SARS-CoV-2 neutralizing antibody REGN10987, which may lead to the failure of neutralization. The results show consistent with the previous experimental conclusion and clarify the structural mechanism under affinity changes. Our methods will offer guidance on the assessment of the infection efficiency and antigenicity effect of continuing mutations in SARS-CoV-2.

## Introduction

Coronavirus disease 2019 (COVID-19), caused by a single-stranded positive-strand RNA virus named severe acute respiratory syndrome-coronavirus 2 (SARS-CoV-2), is a major threat to public health worldwide (Wang et al., 2021). As of 6 June 2021, over 172 million confirmed cases including more than 3.7 million COVID-19 related deaths were reported worldwide according to the World Health Organization.^1^ It has been reported that SARS-CoV-2 infects humans through the binding of the homo-trimeric spike (S) glycoprotein to human angiotensin converting enzyme 2 (hACE2), and this infection mechanism for viral entry is also used by SARS-CoV (Li et al., 2003; Zhou et al., 2020). The surface spike glycoprotein including S1 and S2 subunits is the major antigen of coronaviruses, and S1 binds to host cells whereas S2 mediates viral membrane fusion. The receptor-binding domain (RBD) mediates the binding of the virus to host cells, which is a critical step for viral entry (Hoffmann et al., 2020; Lu et al., 2020; Walls et al., 2020; Zhou et al., 2020). According to the high-resolution crystal structure (Lan et al., 2020; Yan et al., 2020), the receptor-binding motif (RBM) is essential for RBD and contacts highly with hACE2. The structural characterization of the pre-fusion S protein provides atomic level information to guide the design and development of antibody (Wrapp et al., 2020; Wu et al., 2020; Wang et al., 2021).

Neutralizing monoclonal antibodies of the immune system, which play an important role in fighting against viral infections, have been found to target the SARS-CoV-2 RBD and exert neutralization activity by disrupting the virus binding (Cao et al., 2020; Hansen et al., 2020; Ju et al., 2020; Li F. et al., 2020; Liu et al., 2020; Shi et al., 2020). During the virus transmission, alterations of amino acid in the surface spike protein may significantly alter the virus antigenicity and the efficacy of neutralizing antibodies. As SARS-CoV-2 spreads around the world, mutations in spike protein had been continuously reported (Korber et al., 2020; Morse et al., 2020; Saha et al., 2020; Sheikh et al., 2020; van Dorp et al., 2020). There are 930 naturally occurring missense mutations in SARS-CoV-2 spike protein that had been reported in the GISAID database (Supplementary Table 1), and a key mutation from ASP614 to GLY614 (D614G) in SARS-CoV-2 spike protein confer the SARS-CoV-2 more infectious than the original strain (Li Q. et al., 2020). However, most vaccines, testing reagents, and antibodies for SARS-CoV-2 are based on the S protein of the Wuhan reference strain (GenBank: MN908947.3). Among other coronaviruses, missense mutations had been demonstrated to confer resistance to neutralizing antibodies in MERS-CoV and SARS-CoV (ter Meulen et al., 2006; Sui et al., 2008; Tang et al., 2014). In the case of HIV, missense mutations are known to influence envelope glycoprotein expression, virion infectivity (Asmal et al., 2011), alter neutralization sensitivity (LaBranche et al., 2019), and confer resistance with neutralizing antibodies (Bricault et al., 2019; Zhou et al., 2019). The epidemiological observations have proved the mutations, especially in S protein, provide a plausible mechanism for the increased observed infectivity and bring challenges to antibody development for SARS-CoV-2.

Here, we evaluated genomic sequences of SARS-CoV-2 and observed a high frequency (0.72%) mutation occurring in the RBM region, N439K, which was first sampled in March 2020 in Scotland from lineage B.1 on the background of D614G, has arisen independently multiple times. It was observed in 34 countries and the second most commonly observed RBD mutation globally. Study have demonstrated that N439K S protein enhances binding affinity to the hACE2 receptor and reduces the neutralization activity of monoclonal antibodies (Thomson et al., 2021). To further provide insights into the infection and neutralization processes of N439K at the molecular level, we present performed molecular dynamics (MD) simulations of the binary complexes of the RBD domain with the common receptor hACE2 and the neutralizing monoclonal antibody (mAb) CB6/REGN10987, respectively. The structure of N439K-mutated RBD-hACE2 complexes show a new salt bridge and local interaction. The energetic details further indicate that the mutated RBD-hACE2 complexes show higher affinity than the original strain which are mainly attributed to the changes in van der Waals and electrostatic energies of some key residues. On the other hand, N439K reduced the sensitivity to neutralizing antibodies which are mainly attributed to polar solvation and electrostatic interactions. Taken together, the SARS-CoV-2 spike protein with N439K may be more infectious and become resistant to some SARS-CoV-2 neutralizing antibodies.

## Materials and Methods

### Multi-Sequence Alignment and Structure Preparation

In this study, SARS-CoV-2 sequences were aligned against the Wuhan reference genome (GenBank: MN908947.3) using MAFFT (Katoh and Standley, 2013). The wild-type crystal structures, including RBD-hACE2 complex (PDB:6M0J), RBD-CB6 complex(PDB: 7C01) and RBD-REGN10987 complex(PDB: 6XDG), were directly downloaded from the Protein Data Bank^2^ and removed the water molecules using VMD (Humphrey et al., 1996) software. For mutated complexes, firstly wild-type hACE2 and mAbs structures were obtained by removing the RBD structures from RBD-ACE2 and RBD-mAbs complexes, respectively. Then the mutated SARS-CoV-2 RBD (N439K) structures were constructed using homology modeling by SWISS-MODEL (Waterhouse et al., 2018). The mutated SARS-CoV-2 RBD was aligned with hACE2 and mAbs based on the RBD-hACE2 and RBD-mAbs structures using PyMOL software. All structural figures were generated utilizing PyMOL software (Lilkova et al., 2015). Finally, the Ramachandran Plot (Laskowski et al., 1993), ANOLEA (Melo et al., 1997), Z-Score (Pontius et al., 1996), Verify 3D results (Bowie et al., 1991), PROCHECK (Laskowski et al., 1993) and Q-Mean (Waterhouse et al., 2018) method were used to check the quality of model.

### MD Simulation

For each system, MD simulations were performed using GROMACS 5.1.4, the latest CHARMM36 (Tortorici and Veesler, 2019)force field was selected, an explicit solvent model was used by an explicit TIP3P model (Price and Brooks, 2004). Then complexes were solvated in a rectangular periodic box and had a 10 Å buffer distance from along each side, then a suitable number of Na+ and CL- ions were added to neutralize the whole system and mimicked a salt solution concentration of 0.15M. Specifically, the following stages of MD simulations were performed before the production simulation: (1) A full 50,000-step energy minimization was performed with all atoms unrestrained. (2) Each complex was equilibrated with NVT (No. of particles, Volume, and Temperature) ensemble for 1ns. (3) Subsequently, a 50,000,000-step (100 ns) production run was performed at constant pressure (1 bar) and 310K temperature after 1 ns NPT (Wang J. et al., 2020). During the MD simulations, the Particle Mesh Ewald (PME) (Wang et al., 2016) method was applied to account for the long-range electrostatic interactions, and the time step was set to 2 fs in all MD simulations. The salt bridge were calculated using VMD software (Humphrey et al., 1996). Further analyses (RMSD, RMSF, SASA, distances, angles and hydrogen bonds) were performed based on the resulting trajectories by GROMACS tools (Pronk et al., 2013). RMSD shows the deviation from the minimized crystal structure while RMSF shows the deviation from the mean structure over a dynamic ensemble. SASA measures the surface area of a molecule that is accessible to the solvent, generally water, and compares over the course of a simulation to detect solvent exposure events and changes to the protein surface. The SHAKE (Kr?Utler et al., 2015) algorithm was employed to calculate the bonds involving hydrogen atoms, for the H-bond interaction analysis, the two limiting factors are adopted as follows: (1) donor-acceptor distance is ≤ 3.5 Å, and (2) donor-hydrogen-acceptor angle is ≥ 150°.

### MM-PBSA Calculation

Binding free energies of RBDs with hACE2 and mAbs were calculated using g_mmpbsa program. For each binding complex, 200 configurations were taken at an interval of 100 ps from the last 20 ns simulations. The conditions of MM-PBSA calculations are set, including solute dielectric constant = 2, solvent dielectric constant = 80, reference or vacuum dielectric constant = 1 and salt concentration = 0.15. The g_mmpbsa was used to calculate the polar solvation energies, non-polar solvation energies and calculates the free energy of the complex. The general expression of the term is

(1) $$\Delta \,G_{b\,i\,n\,d}=G_{c\,o\,m\,p\,l\,e\,x}-\left(G_{r\,e\,c\,e\,p\,t\,o\,r}+G_{l\,i\,g\,a\,n\,d}\right)$$

Where G_complex_ is the total free energy complex, and G_receptor_ and G_ligand_ are total free energies of the isolated receptor and ligand in solvent, respectively.

(2) $$G_{x}=E_{M\,M}-T\,S+G_{s\,o\,l\,v\,a\,t\,i\,o\,n}$$

Where x is the receptor or ligand or complex. E_MM_ is the average molecular mechanics’ potential energy in a vacuum. TS refers to the entropic contribution to the free energy in a vacuum where T and S denote the temperature and entropy. G_solvation_ is the free energy of solvation.

$$E=E_{b\,o\,n\,d\,e\,d}+E_{n\,o\,n\,b\,o\,n\,d\,e\,d}=E_{b\,o\,n\,d\,e\,d}+\left(E_{v\,d\,W}+E_{e\,l\,e\,c}\right)\,\left(3\right)$$

Where E_bonded_ is bonded interactions consisting of bond, angle, dihedral, and improper interactions. E_nonbonded_ includes both electrostatic and van der Waals interactions, which are depicted using a Coulomb and Lennard-Jones potential function, respectively. In addition, the free energy of solvation has been calculated including polar and nonpolar solvation energies, it that can be depicted as

(3) $$G_{s\,o\,l\,v\,a\,t\,i\,o\,n}=G_{p\,o\,l\,a\,r}+G_{n\,o\,n\,p\,o\,l\,a\,r}$$

Where G_polar_ and G_nonpolar_ are the electrostatic and non-electrostatic contributions to the solvation free energy, respectively. To check the statistical significance between the two complexes, *P*-value was calculated using *t*-test.

## Results

### The Diversity of Mutations in SARS-CoV-2 Whole-Genome Sequence

**FIGURE 1:**
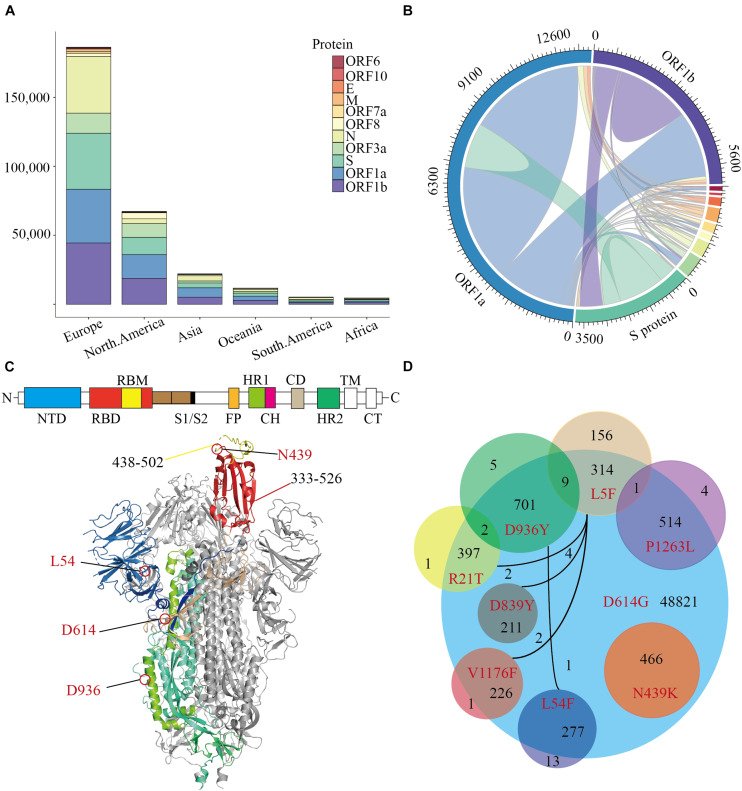
The distribution of Missense Mutations in SARS-CoV-2. A stacked histogram shows all missense mutations of SARS-CoV-2 from different continents of the world. **(B)** A chord diagram shows the missense mutation shared between different proteins of SARS-CoV-2, and the color scheme is the same as that in **(A)**. **(C)** A topology and cartoon representation of SARS-CoV-2 homo-trimeric spike (S) glycoprotein (PDB ID: 6VSB). NTD, N-terminal domain; FP, fusion peptide; HR1, heptad repeat 1; CH, central helix; CD, connector domain; HR2, heptad repeat 1; TM, transmembrane region; CT, cytoplasmic tail; S1/S2, protease cleavage sites; Some important mutation sites and residue intervals were marked on the figure. **(D)** A Venn diagram of main amino acid mutation sites of the S protein, the number of mutations, and co-mutations were shown.

Our trajectory analysis of SARS-CoV-2 mutations in the COVID-19 pandemic was established on 64039 SARS-CoV-2 genome sequences (July 14, 2020) which were downloaded from the Global Initiative for Sharing All Influenza Data (GISAID) database. The SARS-CoV-2 sequences were aligned to the Wuhan reference genome with MAFFT (Katoh and Standley, 2013), then the amino acid changes were identified based on the sequence alignment. In general, most mutations are significantly concentrated in European and North American populations (Figure 1A). When observing the frequency of mutations in all genes, the amino acid changes are mostly distributed in ORF1a, ORF1b, N, and S proteins. Interestingly, the ORF3a protein has a superior mutation rate in North American and Oceanian populations accounting for 14.81 and 10.22% of the total protein, respectively (Figure 1A). The ORF3a protein is also called “accessory protein,” it can convert the environment inside the infected cell and make holes on the infected cell membrane.

The co-mutation may affect the cooperation between the various proteins, which may lead to meaningful virus evolution and pose challenges to the development of antibodies. To investigate the co-mutation in SARS-CoV-2, we calculated the co-mutation in all proteins and found most mutants were not single-site mutations (Figure 1B). Since SARS-CoV-2 infects humans through the binding of trimeric spike glycoprotein (PDB ID: 6VSB) to hACE2, then we counted all amino acid variants on S protein (Figure 1C and Supplementary Table 1). The variant D614G accounts for 75.92% of 64039 SARS-CoV-2 (Korber et al., 2020), following by D936Y accounts for 1.11%. And more notably, N439K is the highest frequency of variant in the RBD region, which accounts for 0.72% of all SARS-CoV-2. Furthermore, there are only 16 mutations on S protein with a mutation rate of more than 0.16% (Supplementary Table 1), and those mutants occur frequently together with the variant D614G (Figure 1D). All the N439K are included in the D614G interestingly, which is mainly concentrated in Europe since March 2020. To conclude, the amino acid changes seem to have been accumulated progressively over time, among which N439K is the most dominant variant in the RBD region and should be preferentially employed to characterize the influence of mutations on pathogen evolution.

### The N439K-Mutated RBD Binds hACE2 With Higher Affinity Than Wild Type

**FIGURE 2:**
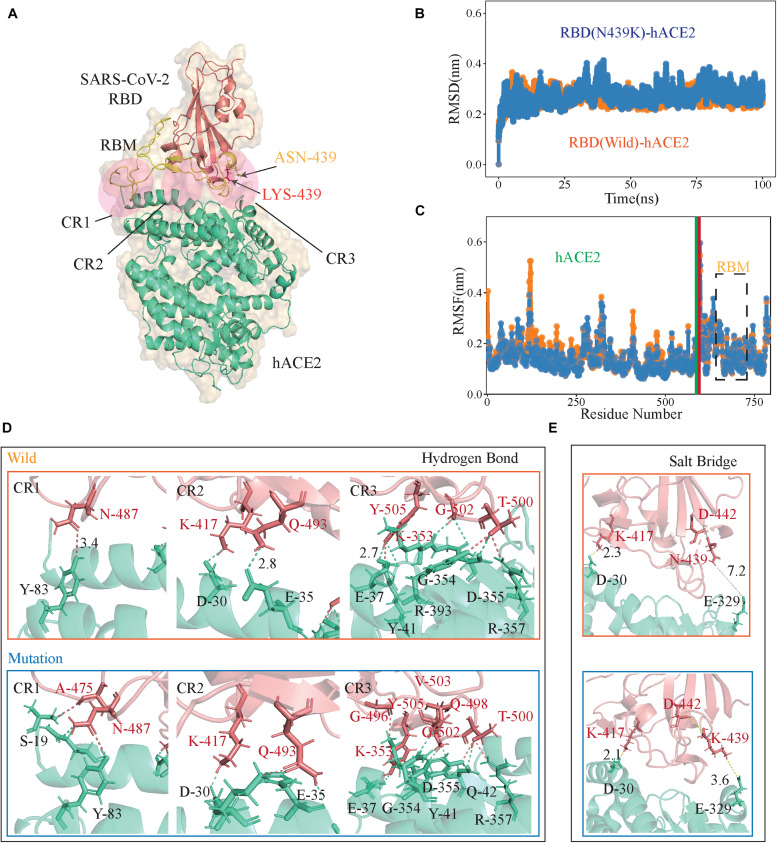
The RBD-hACE2 interaction profile of MD simulations. **(A)** The structure of SARS-CoV-2 RBD-hACE2 (PDB ID: 6M0J). SARS-CoV-2 RBD core is shown in deepsalmon and its interacting hACE2 is colored greencyan, and RBM of RBD is represented in yellow. Receptor-ligand interface includes the N-terminal (CR1), the central region (CR2) and the binding loop (CR3), ASN439 (yellow) to LYS439 (red) in CR3. **(B)** The RMSDs of the backbone atoms of both RBD-hACE2 complexes, the RBD-hACE2 is colored orange and RBD(N439K)-hACE2 is colored blue. **(C)** The RMSFs of *C*_α_atoms of both RBD-hACE2 complexes, where RBD-hACE2 is colored orange and RBD(N439K)-hACE2 is colored blue. **(D,E)** Hydrogen bonds and salt bridges are shown as dotted lines, RBD (deepsalmon), and hACE2 (greencyan) residues are described as sticks, respectively. The orange box indicates wild-type RBD-hACE2 and mutant-type RBD-hACE2 is a blue box.

Crystal structures show that the high contact area of the SARS-CoV-2 RBD-hACE2 complex is mainly concentrated in the RBM region (Shang et al., 2020; Wang Q. et al., 2020), and denoted as CR1, CR2, and CR3 (Figure 2A; Wang Y. et al., 2020). The PROCHECK analysis show both wild-type and N439K S protein model are in good quality, the detail information can be found in Supplementary File 2. To further verify the convergence of MD simulations equilibrium, we estimated the root mean square deviations (RMSD) of backbone atoms relative to the corresponding crystal structure, and the wild complex had a relatively smaller average RMSD than the N439K-mutated complex (2.2 vs. 2.4 Å) (Figure 2B and Table 1). The structural compactness of each system was elucidated by estimating the radius of gyration (R_g_), the average R_g_values were similar for all the same systems (Table 1). We also calculated the solvent accessible surface area (SASA) which indicates the solvent exposure degree, and SASA values of wild and mutant complexes were 35716 Å^2^ and 36121 Å^2^, respectively (Table 1 and Supplementary Table 3). Although the flexibility patterns of residues in the N439K-mutated RBD-hACE2 complex display similar fluctuations with wild-type complex (Figure 2C), certain regions of the two complexes show differences in flexibility.

**TABLE 1:** The average RMSD, solvent accessible surface area (SASA), and radius of gyration for the simulated systems.

**System**	**RMSD (Å)**	**SASA (Å^2^)**	**Rg (Å)**
RBD (wild)-hACE2	2.59(0.23)	35715.96(405.59)	31.58(0.26)
RBD (N439K)-hACE2	2.73(0.34)	36121.38(338.31)	31.54(0.21)
RBD (wild)	2.37(0.39)	10608.14(180.55)	18.23(0.07)
RBD (N439K)	2.76(0.50)	10784.89(155.95)	18.26(0.07)
hACE2 (wild)	2.65(0.23)	26986.65(349.86)	24.98(0.18)
hACE2 (N439K)	3.02(0.65)	27296.22(325.29)	25.14(0.13)

*Standard errors of the mean (SEM) are provided in parentheses.*

Subsequently, we performed the hydrogen bond (H-bond) and salt bridge analyses based on the trajectories in MD simulations. It was observed that the N439K-mutated RBD-hACE2 complex (9.68 ± 3.24) can form more hydrogen bonds than the wild-type (7.26 ± 2.59) during the MD trajectory (P = 2.43 × 10^–138^) (Figure 2D and Supplementary Table 4). Hydrogen bonds from the N-terminal (CR1) to the central region (CR2) of the interface were almost the same during MD simulations, and three additional main-chain hydrogen bonds forms at Gly496, Gln498, and Val503 in the binding loop (CR3), causing the ridge to take a more compact conformation and the loop to move closer to hACE2 (Figure 2D). At the SARS-CoV-2 RBD-hACE2 interface, a strong salt bridge (Pylaeva et al., 2018) between Lys417 of the RBD and Asp30 of hACE2 had been confirmed (Lan et al., 2020; Figure 2E). The mutation of N439K in the RBD formed a new salt bridge with Glu329 of hACE2 (3.6 Å), and a weak salt bridge between Lys439 and Asp442 of SARS-CoV-2 RBD (Figure 2D). Burial of these salt bridges in hydrophobic environments on virus binding would enhance their energy owing to a reduction in the dielectric constant.

**FIGURE 3:**
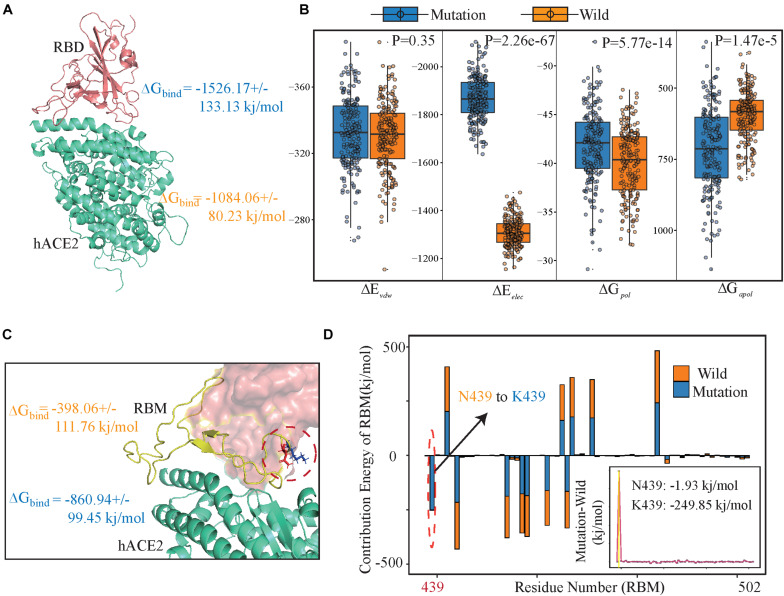
Energy components for binding free energy of both wild and mutated RBD/RBM-hACE2 complexes. **(A)** The binding free energies for SARS-CoV-2 RBD-hACE2 (including wild type and variantN439K) using MM-PBSA. Total binding energy (ΔGbind). **(B)** Energy components for the binding free energy of RBD-hACE2. The intermolecular van der Waals (ΔEvdw); Electrostatic interactions (ΔEelec); Polar solvation energy (ΔGpol) and apolar (non-polar) solvation energy (ΔGapol). **(C)** The binding free energies for SARS-CoV-2 RBM-hACE2 (including wild type and variant N439K), using orange for wild-type and blue for mutant type. **(D)** Decomposition of ΔGbind into contributions from individual residues (438–502) of RBM before and after mutation. All units are reported in kj/mol.

To estimate the stabilization of binding systems, We also calculated the molecular mechanics Poisson-Boltzmann surface area (MM-PBSA) in both wild and mutant SARS-CoV-2 RBD-hACE2 complexes (Kumari et al., 2014). Overall, the binding free energy (ΔG_bind_) decomposition analysis divulged into various free energies (Figure 3B and Supplementary Table 5). The binding energy ΔG_bind_ (−1526.17 ± 133.13 kj/mol) of the N439K-hACE2 was higher in magnitude as compared to the wild-type RBD-hACE2 (−1084.06 ± 80.23kj/mol) (Figure 3A), in which the electrostatic energy between wild and mutant types had a significant difference, which the average ΔE_elec_ (−1876.25 ± 45.23 kj/mol) of the N439K-hACE2 also higher than that of wild-type (−1301 ± 23.14 kj/mol) (Figure 2B). To test the reliability of our simulation, we increase the MD simulation time to 200 ns, the binding energy of 200 ns MD simulation show a similar result, the ΔG_bind_ (−1597.25 ± 179.57 kj/mol) of the N439K-hACE2 also higher than that of wild-type (−959.29 ± 130.36 kj/mol) (Supplementary Table 5). Meanwhile, we calculated binding energy between RBM (residue 438–502) and hACE2 using MM-PBSA, and binding energies of the two complexes were (−860.94 ± 99.45 kj/mol and (−398.06 ± 111.76 kj/mol, respectively (Figure 3C and Supplementary Table 6). Comparing the binding free energy of RBD-hACE2 and RBM-hACE2, it should be noted that the changes in energy are mainly concentrated in the RBM-hACE2 region.

Subsequently, we explored the critical residues involved in the RBM-hACE2 binding by performing the per-residue decomposition of binding free energy (Figure 3D), and the contribution energy of per-residue to the total binding energy was compared before and after mutation. It is evident from the line graph (Figure 3D) that Lys439 hotspot residue show more contribution to the binding free energy than ASN439 and its contribution energy changed from −1.93 to −249.85 kj/mol (Figure 3D). The total binding free energy change between wild-type and RBD-hACE2 (N439K) complexes is 462.88 kj/mol (Figure 3C), mostly concentrated in the electrostatic energy (Figure 3B). Taken together, comparing the interaction interfaces of the N439K-mutated and wild RBD-hACE2 complexes reveals the change from ASN439 to LYS439 might result in a tighter association because of the new salt bridge formation and higher affinity.

### N439K Became Resistant to SARS-CoV-2 Neutralizing Antibody REGN10987

**FIGURE 4:**
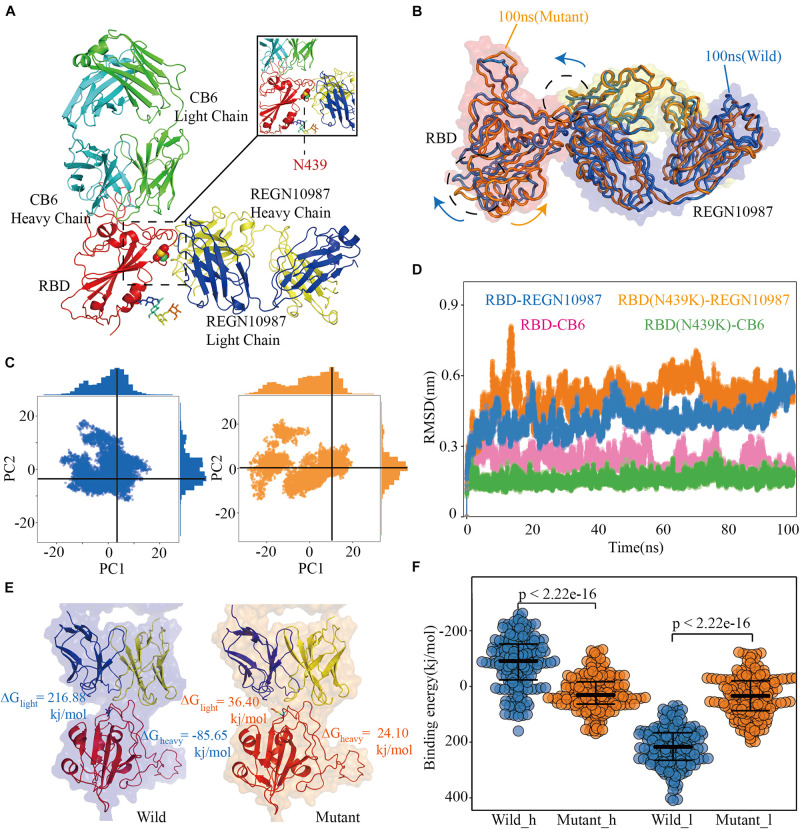
Structural and energetic details of both wild and mutant RBD-mAbs interactions. **(A)** Crystal structures of RDB-CB6/REGN10987 complexes, the RBD is colored red, CB6 heavy and light chains are represented as marine and green, respectively, REGN10987 heavy chain is colored yellow and light is blue, and the 439 residues are described as the sphere. **(B)** Characteristic dynamic fluctuations of both RBD-REGN10987 and RBD(N439K)-REGN10987 complexes. Mutant-type (100 ns) and wild-type (100 ns) are colored by orange and blue, respectively. **(C)** Dynamic conformations are projected on to the principal vectors (PC1 and PC2). Red and blue indicate mutant-type and wild-type 100 ns MD trajectories, respectively. **(D)** The RMSD of the receptor-binding motif in four complexes during the 100-ns MD simulations. **(E)** The binding free energies for both complexes of the mAb REGN10987 (including heavy and light chains), the color schemes are the same in **(A,C)**. **(F)** The binding free energies of 200 configurations at an interval of 100 ps from the last 20 ns simulations. The *t*-test was conducted to check the statistical significance of the difference between two systems of binding free energies. A *p*-value of <0.05 indicates that the difference is statistically significant (95% confidence interval). The color scheme is the same as that in **(C)**.

To investigate the antigenicity of the N439K mutant, we exploited 100 ns MD simulations of the binary complexes of hACE2 with neutralizing monoclonal antibodies REGN10987 and CB6 complexes. Specific neutralizing mAbs can prevent the virus from binding to hACE2 by neutralizing SARS-CoV-2. Although most of these highly binding sites are in RBM, the two neutralizing antibodies show different high contact regions with RBD (Figure 4A).

After the 100 ns MD simulation, the final conformational distribution indicated the configuration fluctuations of RBD were mainly concentrated in the CR3, whereas the REGN10987 antibody was relatively stable (Figure 4B). Overall, SARS-CoV-2 RBD and REGN10987 undergo symmetric twist and antisymmetric hinge-bending motions about the axis of the N-terminal helix, corresponding to the lowest quasi-harmonic models, PC1, and PC2, from principal component analysis (PCA) (Ichiye and Karplus, 1991). Although the overall conformational dynamics were almost the same between the wild and mutant complexes, the average conformation of the variant structure has changed relative to wild complex when the dynamic configurations are projected onto the two principal vectors (Figure 4C). From results of RMSD, the wild and mutant systems had reached a stable state from their respective MD trajectories, CB6 systems had lower average RMSD values than the REGN10987 complexes, and a higher RMSD was calculated in N439K-REGN10987 than wild-type (Figure 4D). The MD simulation results of the CB6-RBD complex can be found in Supplementary Table 7.

To further explain the ability of heavy and light chains of mAbs to neutralize viruses, we had taken 200 structures from the stable region of the last 20ns trajectories to calculate binding free energy with RBD, respectively. It revealed that the estimated binding free energy of wild-type CB6-RBD complexes (−129.73 kj/mol) is higher than N439K-mutated CB6-RBD complexes (−51.28 kj/mol) in the heavy chain, the unfavorable contribution from ΔG_pol_ (491.56 kj/mol) was relatively lower compared to mutant type (703.82 kj/mol) (Table 2). Overall, our research suggested that the N439K reduced the sensitivity to the CB6 mAb. Subsequently, we have investigated the binding free energy of REGN10987-RBD complexes, the ΔG_bind_ of N439K-mutated REGN10987-RBD complexes (24.10 kj/mol) was found to be lower (*P* = 2.22e-16) than wild-type (−85.65 kj/mol) in the heavy chain (Figure 4F and Table 2). Similarly, the light chain had a little difference in binding free energy with RBD, but both systems could not form an effective combination (Figure 4E and Table 2). Furthermore, the present 100 ns trajectory revealed that polar solvation and electrostatic interaction might be the main factors to lose the ability of the REGN10987 antibody to neutralize COVID-19 (Table 2).

**TABLE 2:** Energetic components of binding energy for SARS-CoV-2 RBD-mAbs complexes (kj/mol).

**Systems**	**ΔE_*vdw*_**	**ΔE_*elec*_**	**ΔG_*pol*_**	**ΔG_*apol*_**	**ΔG_*bind*_**
CB6(heavy)-RBD (wild)	−297.09(27.9)	−288.93(67.1)	491.56(112.9)	−35.28(2.5)	−129.73(77.6)
CB6(heavy)-RBD (N439K)	−317.27(18.9)	−400.23(33.6)	703.82(66.3)	−37.59(2.1)	−51.28(67.1)
CB6(light)-RBD (wild)	−140.76(13.1)	29.7(26.3)	217.82(76.8)	−17.77(2.2)	89.03(69.4)
CB6(light)-RBD (N439K)	−123.10(16.4)	82.43(29.5)	205.90(93.7)	−16.76(2.6)	148.46(92.6)
REGN10987(heavy)-RBD (wild)	−135.28(14.9)	−208.45(34.6)	276.61(92.2)	−18.52(2.9)	−85.65(91.0)
REGN10987(heavy)-RBD(N439K)	−105.99(13.3)	−192.56(42.2)	338.70(73.2)	−16.05(2.5)	24.10(61.9)
REGN10987(light) -RBD (wild)	−58.06(9.7)	108.65(60.8)	176.58 (95.0)	−10.28(2.7)	216.88(69.4)
REGN10987(light)- RBD (N439K)	−143.13 (13.0)	55.96(17.0)	142.02 (74.7)	−18.44(2.0)	36.40(71.4)

*Standard errors of the mean (SEM) are provided in parentheses.*

## Discussion

The outbreak of COVID-19 has caused a severe strain on the public health system in many countries. The SARS-CoV-2 virus is expected to continue evolving in human populations. Close monitoring of circulating virus strains is of unquestionable importance to inform research and development of antibodies and therapeutics. Herein, we have studied the mechanism of binding of RBD-hACE2 and RBD-mAbs by using an atomistic molecular dynamics simulation in conjunction with molecular mechanics/Poisson-Boltzmann surface area scheme. Our study shows that the N439K influences the affinity of both RBD-hACE2 and RBD-mAbs complexes which is favored by the intermolecular van der Waals, electrostatic interactions, and polar solvation free energy. Our findings facilitate the development of decoy ligands and neutralizing antibodies for suppression of viral infection.

MD simulations and MM-PBSA results showed that the binding ability of the N439K-mutated RBD with hACE2 was enhanced. In other words, N439K increases spike affinity for ACE2 significantly, which is consistent with the surface plasmon resonance (SPR) result (Thomson et al., 2021). The replacement of ARG426 (SARS-CoV RBD) with ASN439 (SARS-CoV-2 RBD) appeared to weaken the interaction by eliminating one important salt bridge with ASP329 on hACE2 and reduced the affinity (Lan et al., 2020; Yan et al., 2020). The N439K (ASN439 to LYS439) SARS-CoV-2 variant form a new salt bridge at the RBD-hACE2 interface through simulation calculation, which is consistent with the previous study (Thomson et al., 2021). Thomson used SPR to evaluate binding of recombinant N439K RBD protein to recombinant hACE2, indicating that acquisition of the N439K mutation enhances hACE2 binding. Meanwhile, the reduced binding of REGN10987 mAb to the variant N439K RBD, which was also confirmed by bio-layer interferometry analysis (Thomson et al., 2021). Compared with traditional experimental methods, MD simulations require less time, and can explain structure and energy changes at molecular level, such as salt bridge and binging free energy. Further, the highly consistent results with experimental methods verify the power of our method. As the SARS-CoV-2 virus is expected to continue evolving in populations, molecular dynamics simulation is characteristic of high speed and low cost, which is especially suitable for high throughput screening of high-risk mutation in S protein.

A few other circulating RBD mutations have become prominent since N439K first emerged. Independent lineages of SARS-CoV-2 have recently been reported: UK-B.1.1.7, South Africa-B.1.351 and Brail-P.1. The B.1.1.7 variants with increased transmission have 9 amino-acid changes in Spike, including N501Y, and N501Y compromises neutralization by many antibodies with public V-region IGHV3-53 (Supasa et al., 2021). The B.1.351 variants of SARS-CoV-2 containing multiple mutations in Spike are now dominant in South Africa, indicating the potential for impaired efficacy of potential monoclonal antibodies and vaccines (Li et al., 2021). Among the E484K, K417N, and N501Y mutations in the receptor-binding domain of Spike caused widespread escape from monoclonal antibodies (Zhou et al., 2021). The Y435F mutation provides evidence of animal-to-human transmission in mink farms (Oude Munnink et al., 2021). However, these variants with high rates of infection have gained notice, further are studied the impact of own monoclonal antibodies and vaccines. Due to widely spread, it’s difficult to play an early warning role. Whether it is possible to find a way to quickly and effectively monitor the spread of SARS-CoV-2 is still an urgent problem.

In general, vaccinations are being deployed worldwide, which can widely reduce the spread of SARS-CoV-2 among the population. However, as the duration of a SARS-CoV-2 pandemic has prolonged, there have been reported multiple variants around the world. Here, we describe a circulating RBD mutation N439K that maintains a high affinity with hACE2 while evading antibody-mediated immune response using bioinformatics method. More similar reports for SARS-CoV-2 variants have been pointed, and as more and more people develop immune responses against virus via infection or vaccination, the monitoring of SARS-CoV-2 escape mutants is crucial, and may require new vaccine preparations that address the variants circulating globally.

## Data Availability Statement

Publicly available datasets were analyzed in this study. This data can be found here: Publicly available datasets were analyzed in this study. The original sequencing of SARS-CoV-2 can be downloaded from downloaded from the GISAID (https://www.gisaid.org/) database, and the wild-type crystal structure of spike protein binding to ACE2 and mAbs were downloaded from the Protein Data Bank (PDB ID: 6M0J, 6XDG, and 7C01).

## Author Contributions

QJ and HN conceived the project. WZ and CX collected genome sequences and performed multiple sequence alignment. WZ, QJ, CX, PW, ZX, RC, XJ, GX, YG, GX, AA, and LJ contributed to molecular dynamics and binding free energy simulations. QJ, HN, WZ, and CX wrote the manuscript. All authors contributed to the article and approved the submitted version.

## Conflict of Interest

The authors declare that the research was conducted in the absence of any commercial or financial relationships that could be construed as a potential conflict of interest.

## Publisher’s Note

All claims expressed in this article are solely those of the authors and do not necessarily represent those of their affiliated organizations, or those of the publisher, the editors and the reviewers. Any product that may be evaluated in this article, or claim that may be made by its manufacturer, is not guaranteed or endorsed by the publisher.

## Footnotes

1
https://covid19.who.int/

2
https://www.rcsb.org/

## Supplementary Material

Supplementary Table 1 Statistics of the mutations of SARS-CoV-2 from GISAID database.

Supplementary Table 3 Details on both RBD-hACE2 complexes for all simulated systems.

Supplementary Table 4 The number of hydrogen bonds between RBD and hACE2 protein in N439K and wild-type RBD-hACE2 complex.

Supplementary Table 5 Summary on energetic components of binding energy for SARS-CoV-2 RBD-ACE2 complexes.

Supplementary Table 6 Details on energetic components of binding free energy for RBD/RBM-hACE2 complexes.

Supplementary Table 7 Details on binding free energy components for RBD-mAbs complexes.

Supplementary File 2 The quality control results of both wild-type and N439K S protein model.
