# Supplementary material for: N439K Variant in Spike Protein Alter the Infection Efficiency and Antigenicity of SARS-CoV-2 Based on Molecular Dynamics Simulation
Source: Front Cell Dev Biol. 2021 Aug 3;9:697035. doi: 10.3389/fcell.2021.697035 (PMC8369991; doi:10.3389/fcell.2021.697035)
Supplement: Supplementary Table 5 — Summary on energetic components of binding energy for SARS-CoV-2 RBD-ACE2 complexes. [file Table_5.docx]

**Supplementary Table S5. Summary on energetic components of binding energy for SARS-CoV-2 RBD-ACE2 complexes (kj/mol). Standard errors of the mean (SEM) are provided in parentheses.**

| **Systems** | **$\Delta\text{E}_{vdw}$** | **$\Delta\text{E}_{elec}$** | **$\Delta\text{G}_{pol}$** | **$\Delta\text{G}_{apol}$** | **$\Delta\text{G}_{bind}$** |
| --- | --- | --- | --- | --- | --- |
| RBD-ACE2  (100ns, wild) | -330.20(20.40) | -1309.00(59.10) | 595.25(86.99) | -40.11(3.50) | -1084.06 (80.23) |
| RBD-ACE2  (100ns,N439K) | -332.54(21.56) | -1866.42(90.99) | 714.57(147.53) | -41.78( 3.71) | -1526.17(133.13) |
| RBD-ACE2  (200ns, wild) | -314.45 (21.30) | -1126.91(91.13) | 520.96 (138.15) | -38.90(3.82) | -959.29(130.36) |
| RBD-ACE2  (200ns,N439K) | -296.19 (35.64) | -1809.58(70.35) | 545.00 (202.23) | -36.48(4.63) | -1597.25(179.57) |
