# Supplementary material for: N439K Variant in Spike Protein Alter the Infection Efficiency and Antigenicity of SARS-CoV-2 Based on Molecular Dynamics Simulation
Source: Front Cell Dev Biol. 2021 Aug 3;9:697035. doi: 10.3389/fcell.2021.697035 (PMC8369991; doi:10.3389/fcell.2021.697035)

Supplementary File S2

PROCHECK

Wild-type S protein

saves

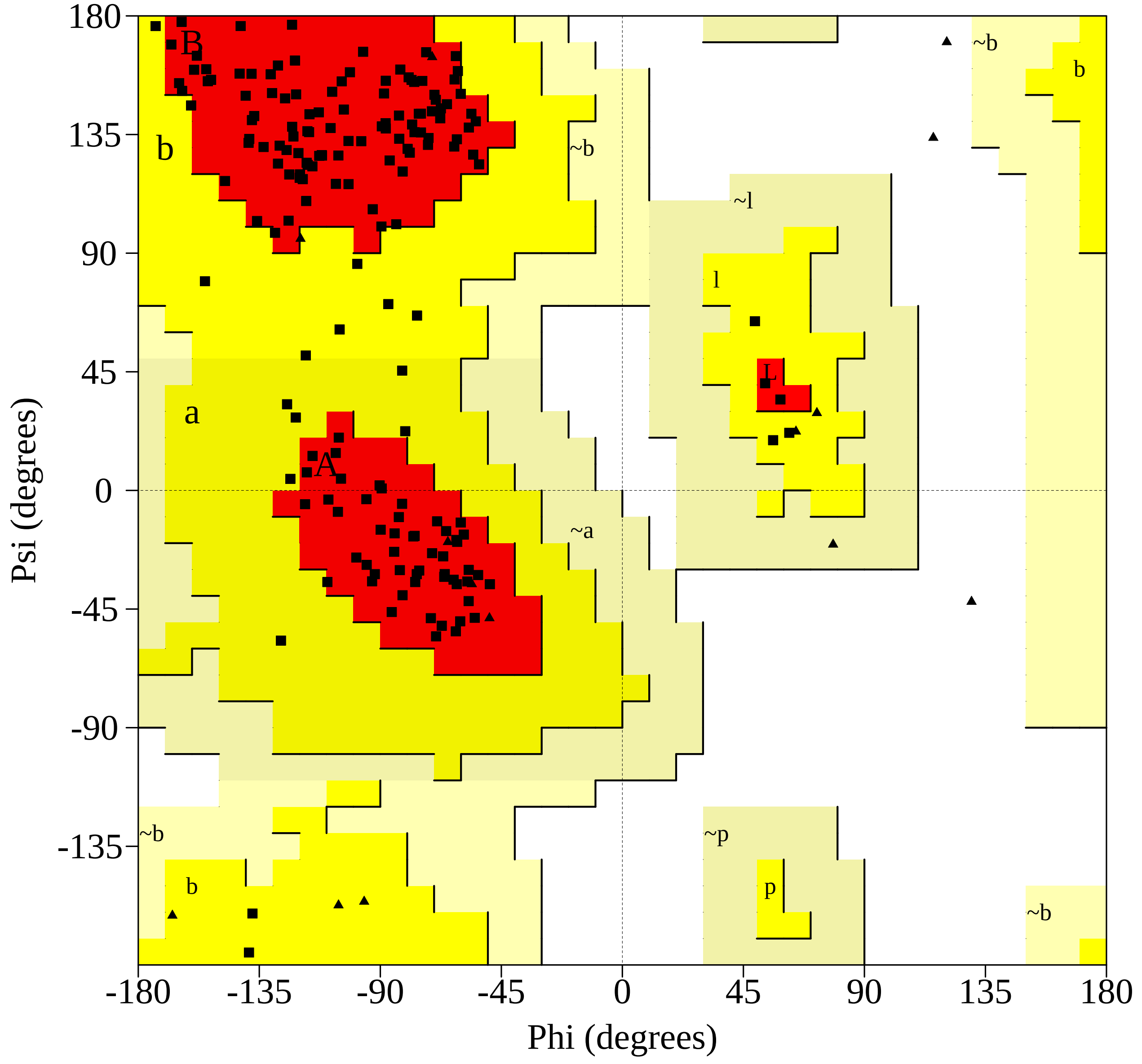

Plot statistics

Residues in most favoured regions [A,B,L]	150	89.3%
Residues in additional allowed regions [a,b,l,p]	18	10.7%
Residues in generously allowed regions [~a,~b,~l,~p]	0	0.0%
Residues in disallowed regions	0	0.0%
	----	------
Number of non-glycine and non-proline residues	168	100.0%
Number of end-residues (excl. Gly and Pro)	1	
Number of glycine residues (shown as triangles)	15	
Number of proline residues	10	

Total number of residues	194	

Based on an analysis of 118 structures of resolution of at least 2.0 Angstroms and R-factor no greater than 20%, a good quality model would be expected to have over 90% in the most favoured regions.

saves_01.ps

PROCHECK

N439K S protein

saves

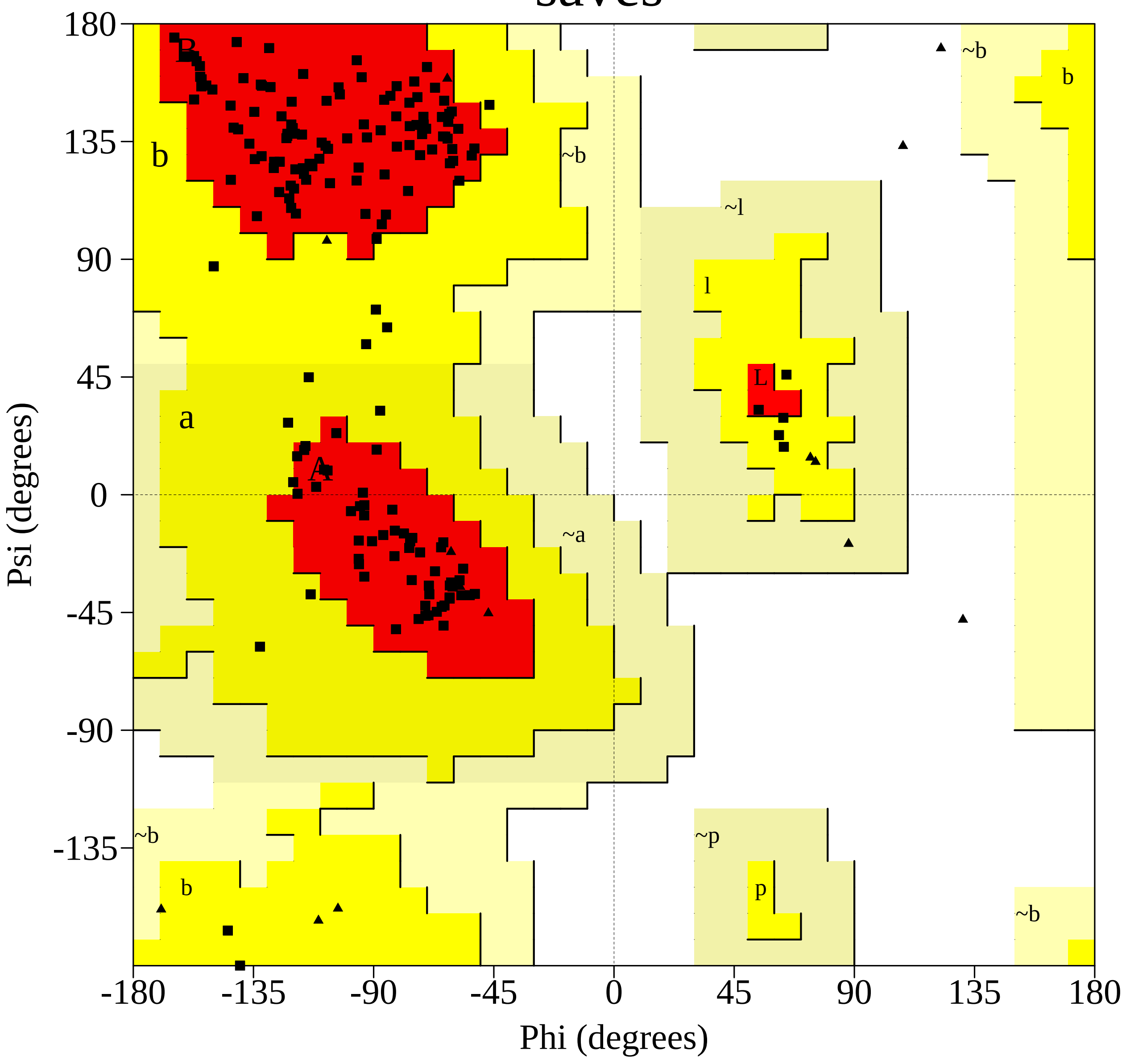

Plot statistics

Residues in most favoured regions [A,B,L]	152	90.5%
Residues in additional allowed regions [a,b,l,p]	16	9.5%
Residues in generously allowed regions [~a,~b,~l,~p]	0	0.0%
Residues in disallowed regions	0	0.0%
	----	------
Number of non-glycine and non-proline residues	168	100.0%
Number of end-residues (excl. Gly and Pro)	1	
Number of glycine residues (shown as triangles)	15	
Number of proline residues	10	

Total number of residues	194	

Based on an analysis of 118 structures of resolution of at least 2.0 Angstroms and R-factor no greater than 20%, a good quality model would be expected to have over 90% in the most favoured regions.

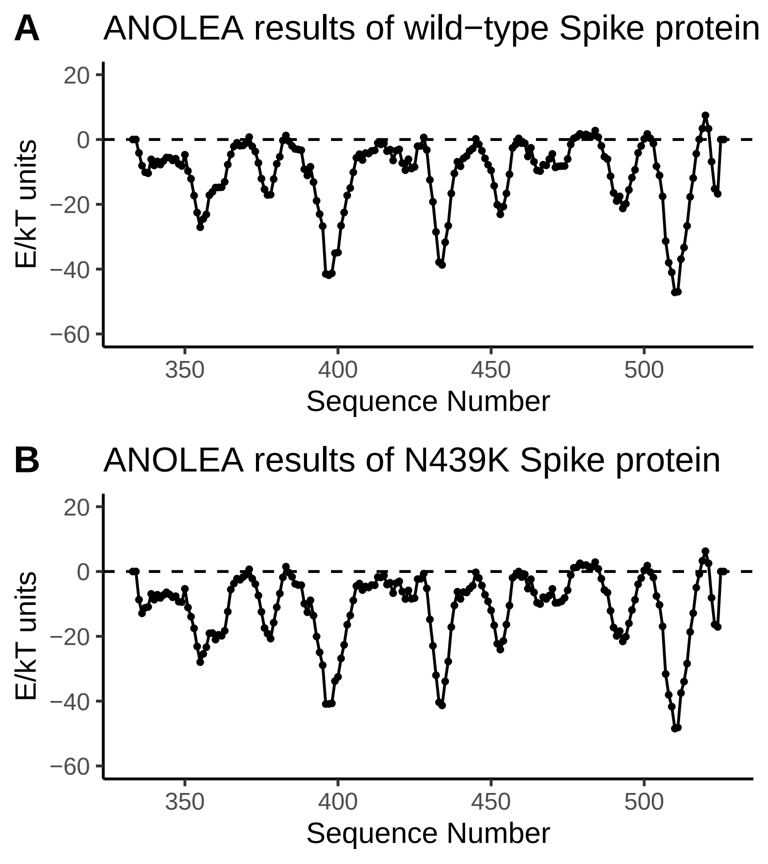
A
ANOLEA results of wild−type Spike protein
E/kT units
20
0
−20
−40
−60
350
400
450
500
Sequence Number
B
ANOLEA results of N439K Spike protein
E/kT units
20
0
−20
−40
−60
350
400
450
500
Sequence Number

Wild-type S protein

Analysis of entire structure

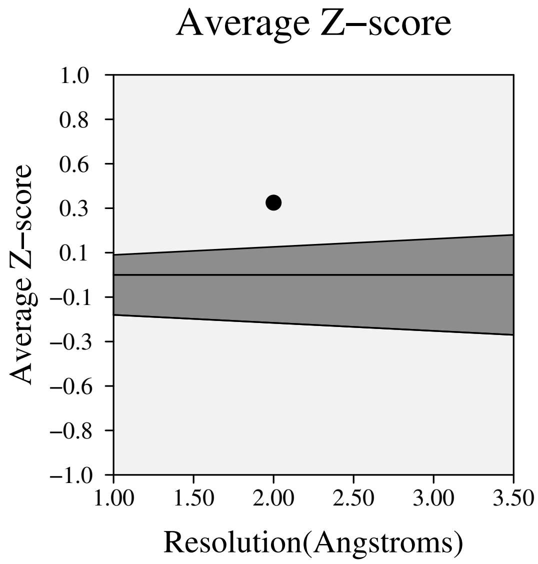

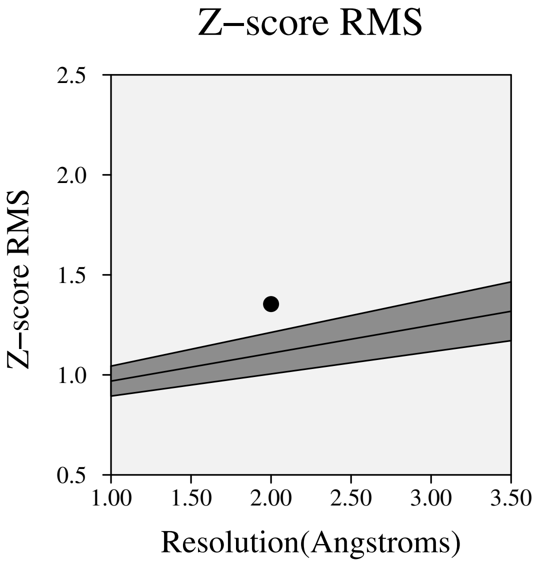

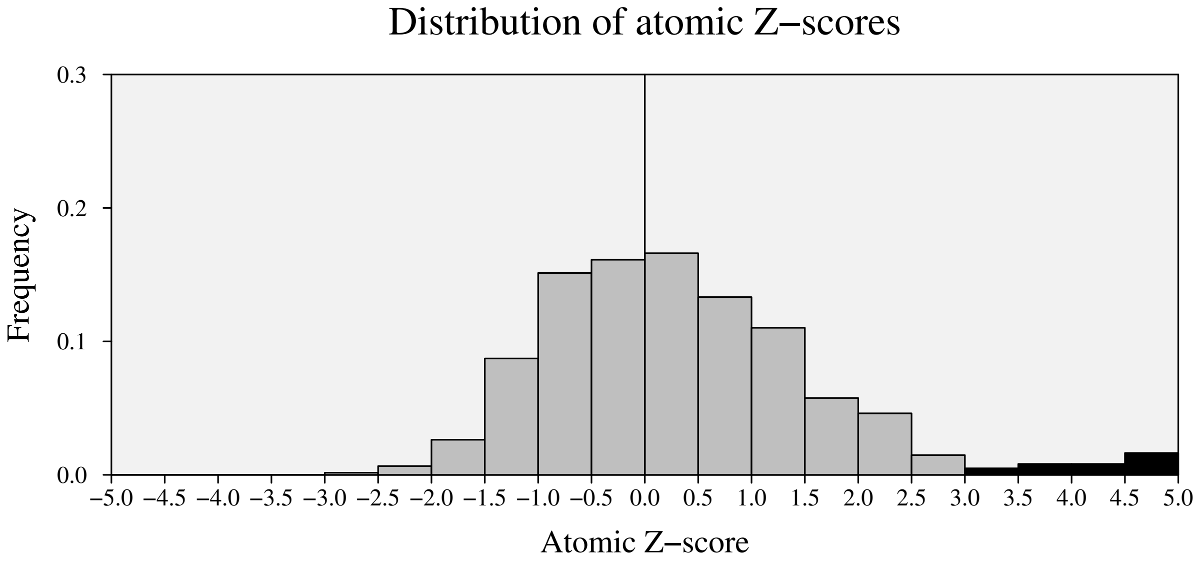

Z−score mean	0.361
Z−score stddev	1.306
Z−score RMS	1.354
# scored atoms	680
# outliers	25
% outliers	3.700

Pontius et al. (1996) J. Mol. Biol. 264, 121−136

N439K S protein

Analysis of entire structure

Average Z−score

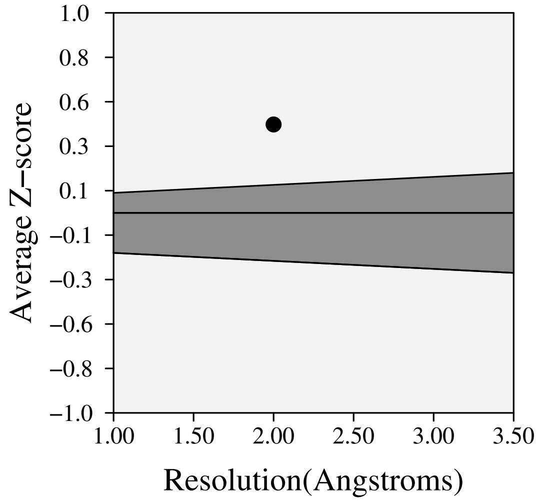

Z−score RMS

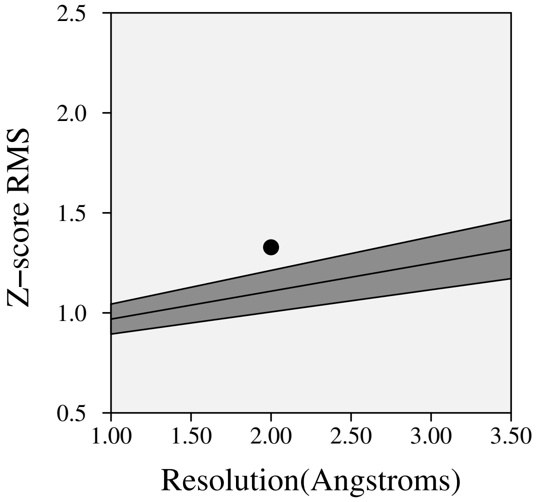

Distribution of atomic Z−scores

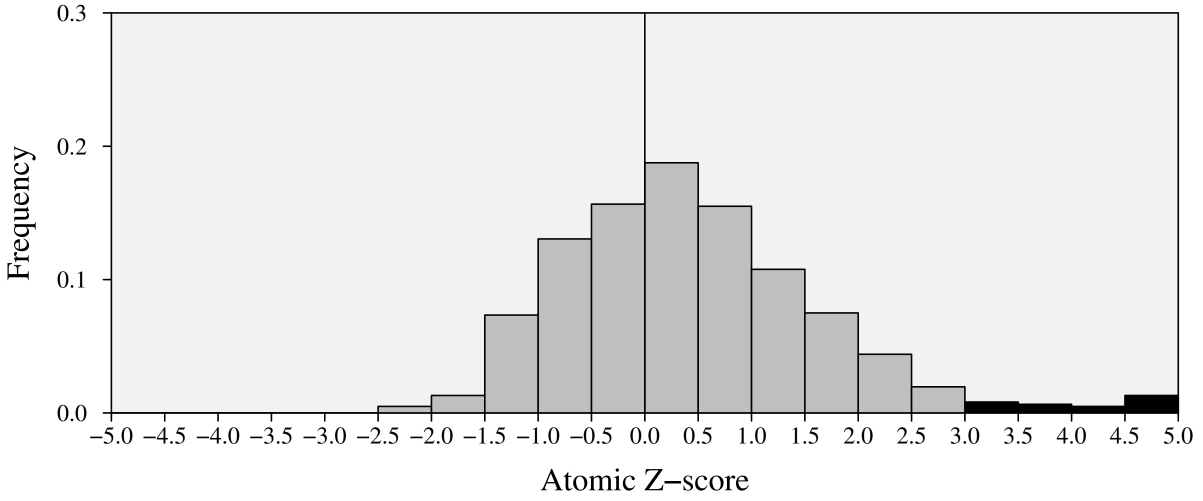

Z−score mean	0.442
Z−score stddev	1.254
Z−score RMS	1.328
# scored atoms	686
# outliers	23
% outliers	3.400

Pontius et al. (1996) J. Mol. Biol. 264, 121−136

A Verify 3D plot of wild-type S protein

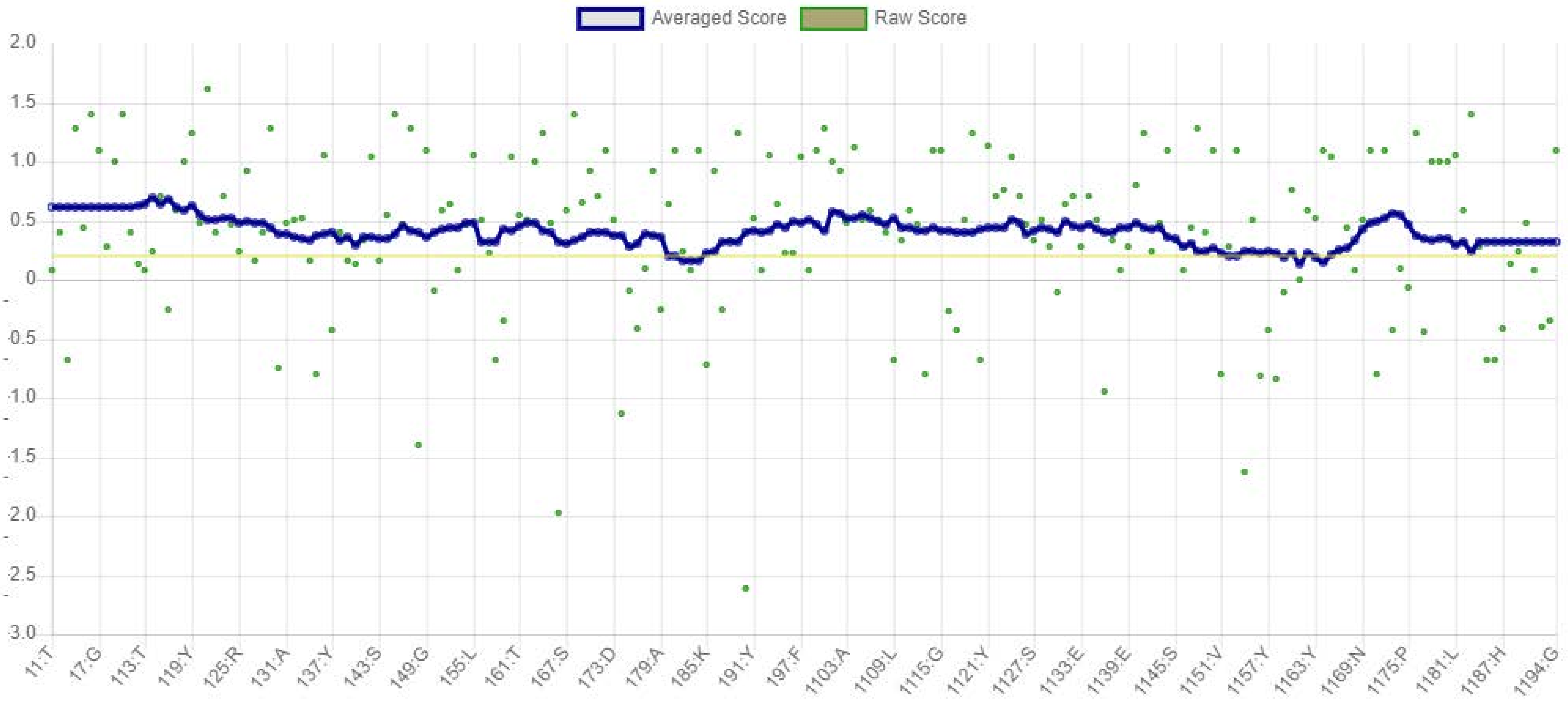

B Verify 3D plot of N439K S protein

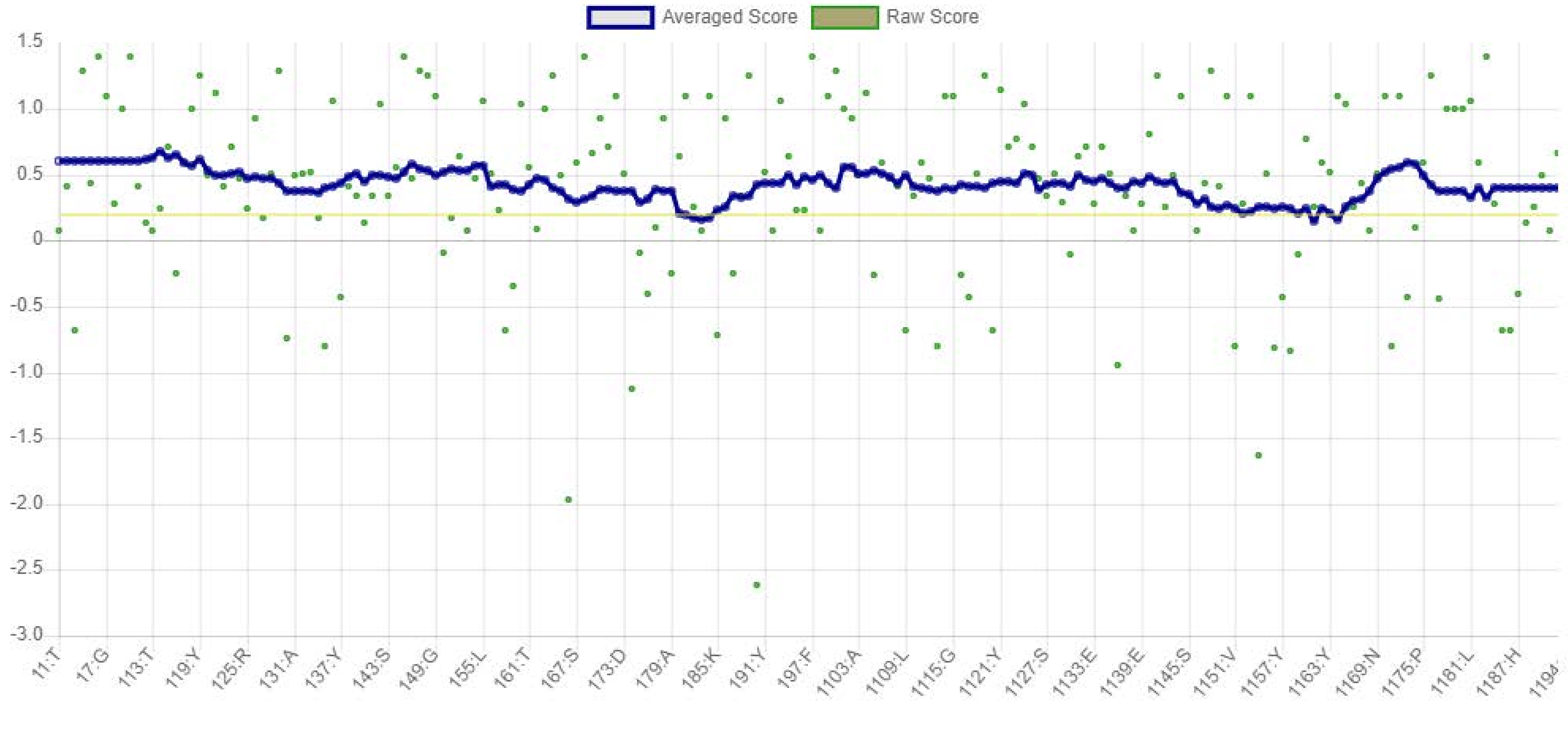

Summary	
Ramachandran plot	**Warning**
All Ramachandrans	**Warning**
Chi1-chi2 plots	**Pass**
Main-chain params	
Side-chain params	**Warning**
Residue properties	**Pass**
Bond len/angle	**Pass**
M/c bond lengths	
M/c bond angles	
Planar groups	**Pass**
Program output	

The PROCHECK of wild-type S protein

```
 +----------<<<  P  R  O  C  H  E  C  K     S  U  M  M  A  R  Y  >>>----------+
 |                                                                              |
 | /var/www/SAVES/Jobs/638906/saves.pdb   1.5                    194 residues |
 |                                                                              |
+| Ramachandran plot:   89.3% core   10.7% allow    0.0% gener    0.0% disall |
 |                                                                              |
+| All Ramachandrans:    2 labelled residues (out of 192)                      |
+| Chi1-chi2 plots:      2 labelled residues (out of 115)                      |
 | Side-chain params:    5 better     0 inside      0 worse                     |
 |                                                                              |
+| Residue properties: Max.deviation:     4.4              Bad contacts:     0 |
 |                     Bond len/angle:    2.2   Morris et al class:  1   2   2 |
 |                                                                              |
 | G-factors           Dihedrals:  -0.27  Covalent:   0.52    Overall:   0.05 |
 |                                                                              |
 | Planar groups:   100.0% within limits   0.0% highlighted                     |
 |                                                                              |
 +------------------------------------------------------------------------------+
   + May be worth investigating further.  * Worth investigating further.
```

Summary file

The PROCHECK of N439K S protein

Summary	
Ramachandran plot	Warning
All Ramachandrans	Warning
Chi1-chi2 plots	Pass
Main-chain params	
Side-chain params	Warning
Residue properties	Warning
Bond len/angle	Warning
M/c bond lengths	
M/c bond angles	
Planar groups	Warning
Program output	

```
 +----------<<<  P  R  O  C  H  E  C  K     S  U  M  M  A  R  Y  >>>----------+
 |                                                                             |
 | /var/www/SAVES/Jobs/638912/saves.pdb   1.5                   194 residues |
 |                                                                             |
 | Ramachandran plot:   90.5% core    9.5% allow    0.0% gener    0.0% disall |
 |                                                                             |
+| All Ramachandrans:    3 labelled residues (out of 192)                     |
+| Chi1-chi2 plots:      2 labelled residues (out of 115)                     |
 | Side-chain params:    5 better     0 inside      0 worse                    |
 |                                                                             |
+| Residue properties: Max.deviation:     4.5              Bad contacts:     0 |
+|                     Bond len/angle:    4.3    Morris et al class:  1  2  2 |
 |                                                                             |
 | G-factors           Dihedrals:  -0.25  Covalent:  -0.04    Overall:  -0.16 |
 |                                                                             |
+| Planar groups:    96.2% within limits   3.8% highlighted       1 off graph |
 |                                                                             |
 +-----------------------------------------------------------------------------+
   + May be worth investigating further.  * Worth investigating further.
```

Summary file

QMEAN of N439K_S_Protein

Model Results

Order by: GMQE

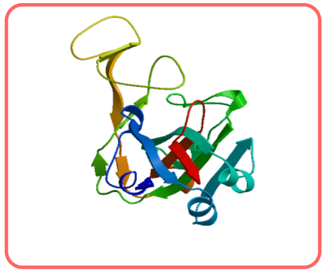

Model 01

Structure Assessment

Oligo-State	Ligands	GMQE	QMEAN
Monomer	None	0.87	-1.66

Global Quality Estimate

QMEAN	-1.66
Cβ	0.17
All Atom	-1.59
solvation	-1.92
torsion	-0.90

Local Quality Estimate

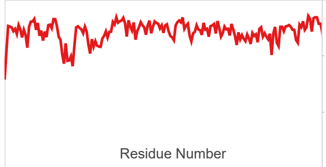

Comparison

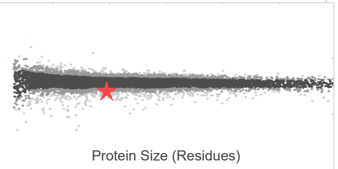

Template	Seq Identity	Coverage	Description
template_upload.1.A	99.48%		Polypeptide

Model-Template Alignment

```
          Model_01 TNLCPFGEVFNATRFASVYAWNRKRISNCVADYSVLYNSASFSTFKCYGVSPTKL  55
template_upload.1.A TNLCPFGEVFNATRFASVYAWNRKRISNCVADYSVLYNSASFSTFKCYGVSPTKL  55

          Model_01 NDLCFTNVYADSFVIRGDEVRQIAPGQTGKIADYNYKLPDDFTGCVIAWNSKNLD 110
template_upload.1.A NDLCFTNVYADSFVIRGDEVRQIAPGQTGKIADYNYKLPDDFTGCVIAWNSNNLD 110

          Model_01 SKVGGNYNYLYRLFRKSNLKPFERDISTEIYQAGSTPCNGVEGFNCYFPLQSYGF 165
template_upload.1.A SKVGGNYNYLYRLFRKSNLKPFERDISTEIYQAGSTPCNGVEGFNCYFPLQSYGF 165

          Model_01 QPTNGVGYQPYRVVVLSFELLHAPATVCG                          194
template_upload.1.A QPTNGVGYQPYRVVVLSFELLHAPATVCG                          194
```

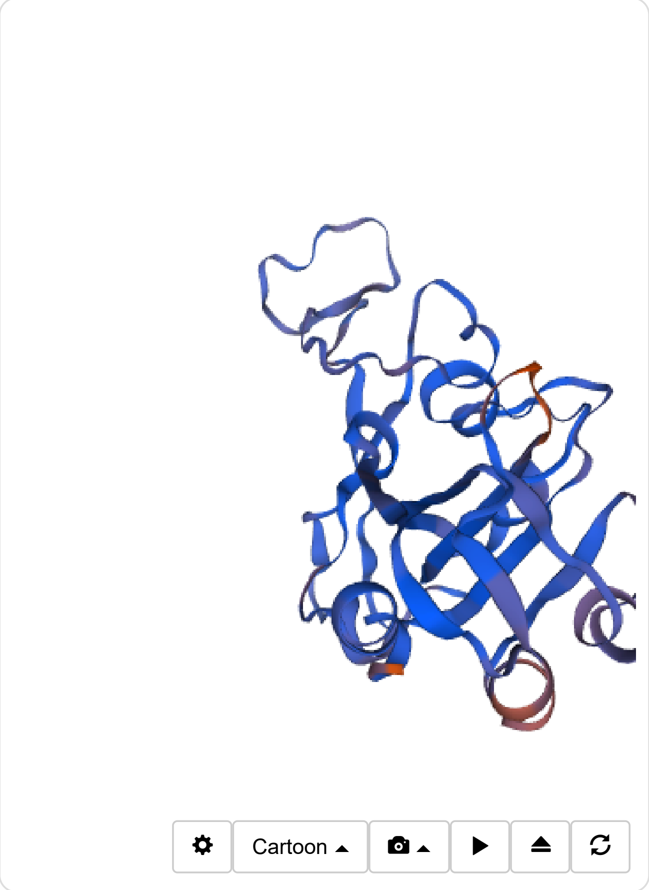

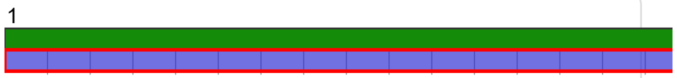

Supplement: Supplementary File 2 — The quality control results of both wild-type and N439K S protein model. [file Data_Sheet_2.pdf]
